# Functional roles and mechanisms of NAT10-mediated RNA ac^4^C modification in normal development and cancer progression

**DOI:** 10.3389/fcell.2026.1795359

**Published:** 2026-03-06

**Authors:** Jie Xiong, Yixiao Yuan, Chongxin Li, Qiao Zhou, Lili Jiang, Xiulin Jiang, Yu Lin

**Affiliations:** 1 Department of Urology, Aerospace Center Hospital, Beijing, China; 2 The First Affiliated Hospital of Chongqing Medical University, Chongqing, China; 3 Department of Oncology, Qujing Central Hospital of Yunnan Province, Qujing, Yunan, China; 4 Department of Radiation Oncology, National Cancer Center/National Clinical Research Center for Cancer/Cancer Hospital, Chinese Academy of Medical Sciences and Peking Union Medical College, Beijing, China

**Keywords:** cancer progression, N4-acetylcytidine, NAT10, physiological function, RNA epitranscriptomics, therapeutic targeting

## Abstract

N-acetyltransferase 10 (NAT10), the primary writer of N4-acetylcytidine (ac^4^C) on RNA, is broadly upregulated across multiple cancer types and correlates with tumor proliferation, invasion, therapeutic resistance, and poor prognosis, indicating its significant clinical relevance. In this mini review, we systematically summarize the functional roles and mechanisms of NAT10-mediated ac^4^C modification in both normal development and cancer. Specifically, we cover recent advances in the regulation of mRNA stability and translation, as well as modifications of tRNA, long non-coding RNA (lncRNA), microRNA (miRNA), and circular RNA (circRNA). Moreover, we integrate evidence supporting the essential roles of NAT10 in embryonic development, gametogenesis, stem cell self-renewal and differentiation, cellular architecture and cell cycle control, and immune cell expansion, while outlining its oncogenic contributions to tumor growth, metastasis, metabolic reprogramming, drug resistance, and immune evasion. Based on clinical findings, we discuss the potential of NAT10 as a diagnostic and prognostic biomarker and its application in liquid biopsy, and we evaluate the therapeutic potential and limitations of targeting NAT10, including toxicity, specificity, and resistance. Finally, we propose future research directions, including the tumor-specific mechanisms driving NAT10 upregulation, strategies for selectively targeting cancer *versus* normal physiology, identification of ac^4^C readers and erasers, and potential crosstalk between ac^4^C and chromatin modification, with the aim of advancing NAT10-based precision oncology.

## 1 Introduction

RNA epigenetic modifications have emerged as a major research focus in recent years (Ye et al., 2023). Analogous to DNA methylation and histone modifications, RNA modifications fine-tune gene expression by altering the chemical structure of RNA molecules, thereby affecting their stability, splicing, translation efficiency, and subcellular localization (Barbieri and Kouzarides, 2020). To date, more than 170 distinct RNA modifications have been identified across various RNA species, among which N6-methyladenosine (m6A) is the most extensively studied (Barbieri and Kouzarides, 2020). However, accumulating evidence indicates that RNA modifications beyond m6A also play critical roles in development, immune responses, and tumorigenesis.

Among these modifications, N4-acetylcytidine (ac^4^C) is a relatively recent addition to the epitranscriptomic landscape. Initially identified in transfer RNA (tRNA) and ribosomal RNA (rRNA), ac^4^C was shown to contribute to RNA stability and translational fidelity (Xie et al., 2023a). With the advent of high-throughput sequencing and modification-specific antibody technologies, ac^4^C has also been detected broadly in messenger RNA (mRNA), where it can markedly enhance mRNA stability and translation efficiency, thereby influencing cell fate decisions and disease progression (Xie et al., 2023a).

Recent studies have identified N-acetyltransferase 10 (NAT10) as the major writer of ac^4^C. As a nucleolar and nucleoplasmic acetyltransferase, NAT10 not only catalyzes RNA acetylation but also regulates cell cycle progression, DNA damage repair, and cellular senescence through multiple mechanisms (Wang et al., 2025a). Importantly, NAT10 is aberrantly upregulated in various cancers and is associated with malignant phenotypes such as increased proliferation, migration, immune evasion, and therapy resistance (Wang et al., 2025a). Consequently, NAT10 has emerged as a promising therapeutic target in oncology. In this mini review, we systematically summarize the functional roles and mechanisms of NAT10-mediated ac^4^C modification in normal development and cancer, and discuss its potential as a therapeutic target and the current progress in this field (Wang et al., 2025a).

## 2 Biological functions of NAT10

**FIGURE 1:**
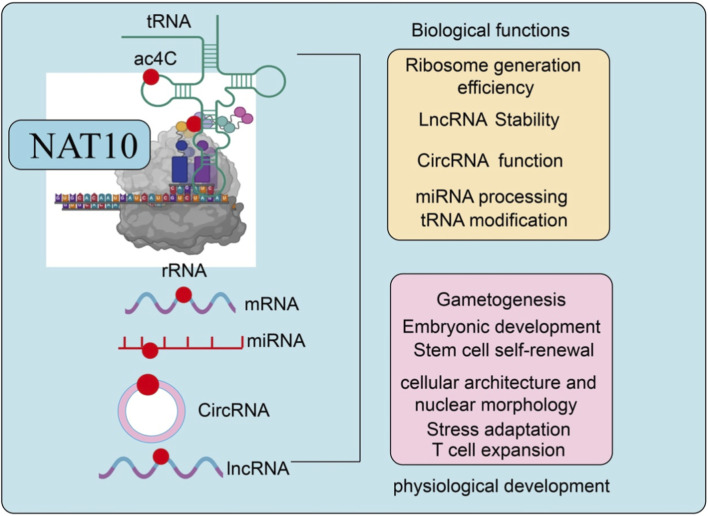
Molecular functions and physiological roles of NAT10-mediated ac4C modification. NAT10 catalyzes ac4C modification on multiple RNA species, including tRNA, rRNA, mRNA, miRNA, circRNA, and lncRNA. Through ac4C modification, NAT10 regulates RNA stability, translation efficiency, ribosome biogenesis, and RNA processing, thereby influencing diverse biological processes such as embryonic development, stem cell self-renewal, stress adaptation, immune cell expansion, and cellular architecture maintenance.

Beyond its oncogenic functions, NAT10-mediated ac4C modification plays a fundamental role in regulating RNA metabolism and cellular homeostasis. As illustrated in Figure 1, ac4C modification orchestrated by NAT10 affects multiple RNA species and is critically involved in diverse physiological and developmental processes.

### 2.1 Regulation of mRNA stability and translation

By catalyzing N4-acetylcytidine (ac^4^C) modification on mRNAs, N-acetyltransferase 10 (NAT10) markedly influences mRNA stability and translation efficiency. Ac^4^C modification enhances the resistance of mRNAs to nuclease-mediated degradation, thereby prolonging mRNA half-life and sustaining gene expression (Wang et al., 2025a). Indeed, the presence of ac^4^C has been shown to significantly stabilize target mRNAs, allowing cells to maintain elevated transcript levels when continuous protein production is required (Li et al., 2025a). In addition, ac^4^C can alter local mRNA secondary structure, facilitating ribosome recognition and recruitment. Compared with unmodified transcripts, ac^4^C-containing mRNAs exhibit increased efficiency during both translation initiation and elongation, leading to higher protein output (Li et al., 2025a). This mechanism is particularly relevant under conditions of rapid proliferation, differentiation, or the high metabolic demands characteristic of cancer cells (Li et al., 2025a). In tumors, NAT10-mediated ac^4^C modification often preferentially targets mRNAs involved in proliferation, migration, anti-apoptosis, and DNA repair, thereby driving malignant phenotypes. For example, mRNAs of key oncogenes display enhanced stability and translation in the context of NAT10 overexpression, promoting tumor growth and therapeutic resistance (Li et al., 2025a). NAT10 functions as the sole known writer of N4-acetylcytidine (ac^4^C) on RNA, and growing evidence indicates that this modification enhances mRNA stability and translation efficiency in a transcript-specific manner (Gu et al., 2024). Mechanistically, ac^4^C modification can increase ribosome occupancy on target mRNAs and promote interactions with RNA-binding proteins that protect transcripts from exonuclease-mediated decay (Li et al., 2025a). High-throughput ac^4^C profiling studies have identified oncogenic mRNAs, such as HMGA1, KRT8, and YAP, as preferential substrates of NAT10, with ac^4^C deposition occurring at defined sequence motifs within coding regions or untranslated regions (Wang et al., 2025a). Functional assays demonstrate that NAT10 depletion reduces ac^4^C levels on these transcripts, leading to decreased mRNA half-life, impaired translation, and consequent attenuation of proliferation, epithelial-mesenchymal transition, or chemoresistance in cancer cells. These findings establish a direct causal link between NAT10 enzymatic activity, post-transcriptional stabilization of specific oncogenic mRNAs, and tumor-promoting phenotypes, underscoring the mechanistic basis for targeting NAT10 in cancer therapy (Wang et al., 2025a).

### 2.2 Regulation of tRNA modification

One of the earliest identified functions of NAT10 is its involvement in tRNA modification. As a central component of protein synthesis, tRNA modification directly affects translational accuracy and efficiency (Zhang et al., 2025). In tRNAs, ac^4^C modification stabilizes the anticodon loop structure and improves correct aminoacyl-tRNA recognition and pairing, thereby reducing translation errors and enhancing the fidelity of protein synthesis (Zhang et al., 2025). Under stress conditions such as nutrient deprivation or oxidative stress, dynamic changes in tRNA modification are part of the cellular adaptive response. By regulating ac^4^C levels on tRNAs, NAT10 contributes to cellular adaptation and survival under environmental challenges. Since tumor cells generally require high translational capacity, altered tRNA modification may support increased protein synthesis and thus facilitate rapid proliferation (Zhang et al., 2025). In this context, NAT10 overexpression in cancers may promote translational adaptation and metabolic reprogramming by elevating tRNA ac^4^C modification.

### 2.3 Regulation of lncRNA expression

Long non-coding RNAs (lncRNAs) play critical roles in gene regulation, and their functions are influenced by expression level, splicing, and structural conformation. Increasing evidence suggests that NAT10 enhances lncRNA stability via ac^4^C modification (Wang et al., 2025b). Similar to mRNAs, ac^4^C marks on lncRNAs can prolong transcript half-life, leading to increased expression and regulatory activity. For instance, in gastric cancer, NAT10 stabilizes lncRNA XIST through ac^4^C modification, which subsequently recruits hnRNPK to promote YAP1 nuclear translocation and activate TEAD4-dependent VEGFA transcription, thereby driving abnormal angiogenesis (Lei et al., 2025). Inhibition of NAT10 can improve vascular structure and reshape the immune microenvironment, enhancing cytotoxic lymphocyte infiltration while reducing regulatory T cells (Tregs), thus improving the efficacy of immune checkpoint blockade (Lei et al., 2025). In esophageal squamous cell carcinoma, NAT10-mediated ac^4^C modification stabilizes lncRNA CTC-490G23.2, sustaining its upregulation in both primary and metastatic tissues (Yu et al., 2023). As a molecular scaffold, CTC-490G23.2 facilitates PTBP1-mediated alternative splicing of CD44, shifting from CD44s to the pro-metastatic CD44v (8–10) isoform, which stabilizes vimentin and promotes tumor invasion and metastasis (Yu et al., 2023). In our recent work, we found that NAT10-mediated ac^4^C modification upregulates LINC02802, which functions as a competing endogenous RNA (ceRNA) for miR-1976. By sponging miR-1976, LINC02802 relieves its repression of solute carrier family 25 member 51 (SLC25A51). Elevated SLC25A51 enhances mitochondrial NAD^+^ import, increasing the NAD^+^/NADH ratio and promoting oxidative tricarboxylic acid (TCA) cycle flux (Yuan et al., 2025).

### 2.4 Regulation of miRNA processing

MicroRNAs (miRNAs) are key post-transcriptional regulators, and their biogenesis involves multiple steps including transcription, Drosha/DGCR8-mediated cleavage, nuclear export, and Dicer processing (Zhang et al., 2024a). NAT10 may affect the stability and processing efficiency of primary or precursor miRNAs (pri-miRNAs/pre-miRNAs) through ac^4^C modification (Zhang et al., 2024a). By stabilizing miRNA precursors, NAT10 promotes the production of mature miRNAs. For example, recent studies have shown that NAT10, together with THUMPD1, introduces ac^4^C into pri-miRNAs, enhancing their interaction with DGCR8 and thereby facilitating pri-miRNA processing into precursor miRNAs (Zhang et al., 2024a). As NAT10 is highly expressed in various cancers and correlates with poor prognosis, its knockdown impairs miRNA biogenesis and attenuates oncogenic phenotypes in lung cancer cells, suggesting that targeting NAT10-mediated pri-miRNA ac^4^C modification may represent a promising anticancer strategy.

### 2.5 Regulation of circRNA function

Circular RNAs (circRNAs) are highly stable due to their covalently closed structure and can act as miRNA sponges, protein scaffolds, or even translation templates (Zhang et al., 2024b). Circular RNAs (circRNAs) are emerging regulators of gene expression, often acting through interactions with RNA-binding proteins (RBPs) or modulation of mRNA stability. CircRNAs, highly stable and conserved RNAs capable of encoding functional peptides, play key roles in cellular processes and cancer progression, and hold potential as biomarkers and therapeutic targets (Khan et al., 2022a; Khan et al., 2022b). m6A is a prevalent internal mRNA modification that dynamically regulates gene expression by influencing mRNA splicing, export, translation, stability, and non-coding RNA function, and its dysregulation is linked to a range of human diseases—including cancer, cardiovascular, neurodegenerative, and liver disorders—highlighting its potential as both a biomarker and therapeutic target. Recent evidence indicates that NAT10-mediated ac^4^C modification extends beyond mRNAs to be influenced by circRNAs. For example, in cervical cancer, circMAST1 suppresses tumor growth and metastasis by modulating the NAT10/YAP axis (Zhang et al., 2024b). Mechanistically, circMAST1 directly binds NAT10 and prevents its association with YAP mRNA, thereby reducing ac^4^C modification on YAP transcripts, decreasing their stability, and ultimately lowering YAP protein levels (Zhang et al., 2024b). This post-transcriptional regulation attenuates YAP-driven proliferation and lymph node metastasis. These findings suggest that circRNAs can act as endogenous modulators of NAT10 activity, potentially by sequestering the enzyme or altering its substrate accessibility, and highlight a novel layer of circRNA-mediated control over ac^4^C-dependent mRNA regulation (Zhang et al., 2024b). CircRNAs have long been considered non-coding RNAs. However, emerging studies have revealed that certain circRNAs possess translational potential under specific conditions (Lan et al., 2025). Lacking a 5′cap and poly(A) tail, circRNAs cannot recruit ribosomes via canonical cap-dependent mechanisms. Notably, m^6^A modifications can act as IRES-like elements on circRNAs, directly facilitating ribosome assembly and translation initiation. Even a single or few m^6^A sites can significantly enhance circRNA translation, in a manner dependent on sequence context and RNA secondary structure (Lan et al., 2025). m^6^A-mediated local structural alterations and recruitment of m^6^A readers enable translation initiation complexes to engage circRNAs without a 5′cap, conferring cap-independent translational competence (Lan et al., 2025). This mechanism is particularly important under stress conditions or tumor microenvironmental perturbations, allowing rapid production of specific functional peptides (Lan et al., 2025). Increasing evidence indicates that circRNA-encoded peptides participate in tumor cell proliferation, invasion, metastasis, metabolic reprogramming, and therapy resistance (Margvelani et al., 2025). These peptides may act as modulators of oncogenic pathways such as PI3K/AKT, MAPK, or Wnt/β-catenin, or influence protein stability and subcellular localization through direct interactions. Furthermore, due to tissue- and disease-specific circRNA expression, these peptides are limited in normal tissues, highlighting their potential in tumor-specific therapy and immunomodulation.

At the molecular level, m^6^A-driven circRNA translation relies on specific readers and translation initiation factors. YTHDF3 serves as a critical bridge linking m^6^A marks to the translational machinery, specifically recognizing m^6^A sites on circRNAs and recruiting the translation initiation factor eIF4G2 (DAP5) (Yang et al., 2017). eIF4G2 facilitates ribosome assembly and initiation independently of the cap-binding protein eIF4E. Through the m^6^A–YTHDF3–eIF4G2 axis, circRNAs achieve efficient translation of functional peptides in a cap-independent manner (Yang et al., 2017). In tumor cells, this axis is often aberrantly activated, leading to sustained expression of oncogenic circRNA-derived peptides and promoting tumor progression or immune evasion. Therefore, the m^6^A–YTHDF3–eIF4G2 pathway not only reveals a novel mechanism of circRNA translation regulation but also provides a theoretical basis for targeting RNA modifications or circRNA-encoded products in anticancer strategies (Yang et al., 2017).

### 2.6 How NAT10 recognizes and acetylates diverse RNA substrates

NAT10 is currently the only identified enzyme responsible for catalyzing ac4C)deposition on RNA, acting on diverse substrates including mRNA, tRNA, and ribosomal RNA (Wang et al., 2025a). Its enzymatic activity is dependent on acetyl-CoA as the acetyl donor and ATP as an energy source, enabling the transfer of an acetyl group to the exocyclic N4 position of cytidine in an ATP-dependent manner (Chen et al., 2025a). Emerging evidence suggests that NAT10 does not recognize all RNA substrates autonomously but instead relies on auxiliary factors to achieve substrate specificity. For example, THUMPD1 in mammalian cells and its yeast homolog Tan1 have been identified as adaptor proteins that facilitate NAT10-mediated tRNA acetylation, whereas modification of 18S rRNA requires small nucleolar RNAs such as human SNORD13 or yeast U13, which guide NAT10 to specific cytidine residues through extensive base-pairing interactions reminiscent of snoRNA-guided rRNA modification mechanisms (Chen et al., 2025a). Structurally, NAT10 contains a conserved acetyltransferase domain responsible for catalysis, together with RNA-binding regions that enable interactions with RNA substrates and guiding cofactors, thereby supporting its dual capacity to acetylate both RNA and protein targets. Notably, while NAT10 is firmly established as the ac4C “writer,” no dedicated eraser for ac4C has yet been identified (Xie et al., 2023a). Motif analysis revealed that both ac4C modification sites and NAT10 binding peaks were significantly enriched for CU-containing sequences. Recent studies further extend the substrate spectrum of NAT10 to non-coding RNAs, particularly long non-coding RNAs and primary microRNAs (Chen et al., 2025a). Acetylation of specific lncRNAs has been shown to enhance their stability and expression in cancer and neurodegenerative disease contexts, while ac4C modification of pri-miRNAs promotes their processing into precursor miRNAs, contributing to tumor initiation and progression. However, the precise molecular mechanisms by which ac4C influences lncRNA turnover or miRNA biogenesis remain largely undefined, underscoring important directions for future investigation (Wang et al., 2025a).

## 3 Functions and mechanisms of NAT10 in normal physiological development

As a key RNA acetyltransferase, N-acetyltransferase 10 (NAT10) participates in diverse physiological processes, particularly embryonic development, gametogenesis, stem cell fate determination, and the maintenance of cellular architecture and nuclear morphology (Xiao et al., 2024). Its functions extend beyond ac^4^C-mediated regulation of RNA stability and translation, encompassing nucleolar integrity, nuclear scaffold organization, and stress response pathways.

### 3.1 Embryonic development

NAT10 plays a pivotal role during embryogenesis, primarily by modulating gene expression programs through RNA acetylation. By enhancing mRNA stability and translational efficiency, NAT10 promotes the expression of developmentally important proteins, thereby supporting rapid proliferation and organogenesis (Zhang et al., 2024c). In addition, NAT10 contributes to genome stability by participating in DNA damage repair, reducing the accumulation of mutations during development and ensuring normal embryonic progression. For instance, ac^4^C modification of mRNA is markedly increased during human embryonic stem cell (hESC) differentiation toward the ectoderm, with particularly high enrichment in neural epithelial progenitors (NEPs) (Zhang et al., 2024c). Mechanistically, NAT10-mediated ac^4^C enhances the translation of NR2F1 mRNA, which in turn facilitates YAP1 nucleo-cytoplasmic translocation, thereby promoting ectodermal differentiation. These findings indicate that ac^4^C can regulate embryonic cell fate by boosting the translation of key developmental transcription factors (Zhang et al., 2024c). Recent studies provide direct genetic and mechanistic evidence that NAT10 is essential for early embryonic development. Using Nat10 siRNA microinjection and growing oocyte–specific Nat10 knockout mice (Zp3-Nat10^lox/lox^), NAT10 was shown to be indispensable for zygotic genome activation (ZGA) and the morula-to-blastocyst transition. Loss of NAT10 caused embryonic arrest at the 2-cell or morula stage, accompanied by impaired maternal mRNA clearance and defective ZGA (Cui et al., 2025). Mechanistically, NAT10-mediated ac^4^C modification promotes the stability of key developmental transcripts, such as Nanog, and regulates the expression of lineage specification factors including NANOG and CDX2 (Cui et al., 2025). In addition, NAT10-mediated ac^4^C enhances translation efficiency of specific developmental mRNAs during early lineage commitment, as exemplified by acetylation of NR2F1 mRNA in human embryonic stem cells to promote ectoderm differentiation (Ge et al., 2024). Together, these findings establish NAT10 as a maternal-effect regulator of early embryogenesis through ac^4^C-dependent control of mRNA stability and translation of key developmental regulators.

### 3.2 Gametogenesis

During gametogenesis, NAT10 contributes to both spermatogenesis and oogenesis by regulating RNA modification and translational processes (Jiang et al., 2023). NAT10-mediated ac^4^C modification enhances the stability of RNAs involved in germ cell development, promoting the expression of proteins essential for differentiation and maturation (Jiang et al., 2023). By modulating protein synthesis and cell cycle progression, NAT10 supports germ cell differentiation and maturation. NAT10 may also influence chromosomal organization through its role in nuclear structure and nucleolar function, thereby ensuring proper formation of sperm and oocytes. Notably, NAT10 is the only known ac^4^C “writer” and has been shown to be indispensable during murine spermatogenesis, with dynamic changes in mRNA ac^4^C levels across different developmental stages (Jiang et al., 2023). Germ cell-specific deletion of Nat10 blocks the entry into meiosis and leads to defects in homologous chromosome synapsis, recombination, and DNA double-strand break repair. These results suggest that ac^4^C supports spermatogenesis by maintaining the homeostasis of meiosis-related gene expression (Jiang et al., 2023). Recent genetic studies provide direct evidence for an essential role of NAT10 in gametogenesis and early developmental competence. Oocyte-specific deletion of Nat10 abolishes ac^4^C deposition on maternal mRNAs and results in severe meiotic maturation defects and female infertility (Chen et al., 2025a). Mechanistically, loss of NAT10-mediated ac^4^C reduces the translation efficiency of key maternal transcripts involved in mRNA stability and the maternal-to-zygotic transition (Chen et al., 2025a). In the male germline, germ cell–specific Nat10 ablation disrupts meiotic entry, homologous chromosome synapsis, meiotic recombination, and DNA double-strand break repair, accompanied by widespread dysregulation of meiotic genes (Chen et al., 2022). Together, these findings establish NAT10 as a critical regulator of gametogenesis through ac^4^C-dependent control of developmental mRNA translation and meiotic progression.

### 3.3 Stem cell self-renewal and differentiation balance

NAT10 is essential for maintaining stem cell self-renewal by regulating the stability and translation of mRNAs encoding key pluripotency factors (Liu et al., 2023). During differentiation, changes in NAT10 expression and activity can alter the ac^4^C modification landscape of differentiation-related transcripts, thereby promoting or inhibiting specific lineage commitment. Through dynamic RNA acetylation, NAT10 enables the preservation of stem cell pools while supporting timely differentiation responses (Liu et al., 2023). Emerging evidence indicates that NAT10 regulates stem cell self-renewal and lineage commitment in multiple stem cell systems, including embryonic and hematopoietic stem cells (Liu et al., 2023). In human embryonic stem cells (hESCs), NAT10 expression positively correlates with pluripotency both *in vivo* and *in vitro*. Transcriptome-wide ac^4^C profiling revealed enrichment of ac^4^C modifications on mRNAs encoding core pluripotency transcription factors (Liu et al., 2023). Genetic inactivation of NAT10 reduced ac^4^C levels on target transcripts, accelerated decay of the pluripotency regulator OCT4 (POU5F1) mRNA, impaired self-renewal, and promoted premature differentiation, demonstrating a direct role of NAT10-mediated ac^4^C in maintaining stemness through mRNA stabilization (Liu et al., 2023).

In the hematopoietic system, Nat10-mediated ac^4^C modification is essential for hematopoietic stem and progenitor cell (HSPC) maintenance and differentiation. Using low-input ac^4^C mapping in rare HSPCs, dynamic and cell-type–specific ac^4^C landscapes were identified, with Nat10 expression and ac^4^C levels peaking in megakaryocyte–erythroid progenitors. Nat10 knockout disrupted HSC self-renewal and arrested megakaryocyte–erythroid differentiation, leading to hematopoietic failure (Huang et al., 2026). Mechanistically, NAT10 deposits ac^4^C on mRNAs encoding key hematopoietic transcription factors, such as Nfix, thereby enhancing their translation; restoration of NFIX expression partially rescued differentiation defects in Nat10-deficient HSPCs (Huang et al., 2026). Together, these studies establish NAT10 as an epitranscriptomic regulator of stem cell fate through ac^4^C-dependent control of mRNA stability and translation. Nevertheless, whether similar mechanisms operate in other adult stem cell systems remains to be determined.

### 3.4 Regulation of cellular architecture and nuclear morphology

NAT10 also contributes to the maintenance of nucleolar structure and function. It is involved in rRNA processing and ribosome biogenesis, which are essential for nucleolar integrity (Larrieu et al., 2014). Dysfunction or loss of NAT10 can lead to nuclear morphological abnormalities, such as nuclear envelope irregularities and nuclear condensation, ultimately impairing cellular function (Larrieu et al., 2014). By regulating nuclear-cytoskeletal connections and nuclear membrane protein expression, NAT10 participates in maintaining cell shape and tissue organization. Recent studies identified two nuclear/nucleolar localization signals in NAT10 (residues 68–75 and 989–1,018); deletion of these motifs causes NAT10 translocation from the nucleolus to the cytoplasm and plasma membrane (Shen et al., 2009), where it colocalizes with α-tubulin and integrin. Cytoplasmic/plasma membrane-localized NAT10 promotes α-tubulin acetylation and microtubule stabilization, thereby enhancing migration and invasion of hepatocellular carcinoma cells (Shen et al., 2009). NAT10 plays a critical role in maintaining cellular architecture, encompassing nuclear morphology, cytoskeletal organization, and cell cycle progression (Larrieu et al., 2014). NAT10 localizes predominantly to the nucleolus, where it regulates nuclear shape by modulating chromatin organization and nucleolar structure (Larrieu et al., 2014). Beyond the nucleus, NAT10 acetylates cytoskeletal components, including microtubules, thereby influencing spindle assembly and mitotic progression (Larrieu et al., 2018). Pharmacological inhibition of NAT10 with Remodelin disrupts these processes, leading to aberrant nuclear morphology, altered microtubule dynamics, delayed mitotic progression, and impaired chromosomal segregation (Larrieu et al., 2018). Collectively, these findings highlight NAT10 as a central regulator of structural homeostasis, coordinating nuclear integrity and cytoskeletal function to ensure proper cell division and genome stability.

### 3.5 Stress adaptation and developmental homeostasis

During development, cells face environmental and metabolic stressors, and NAT10 contributes to adaptive responses through multiple mechanisms (Fu et al., 2025). First, NAT10 participates in DNA damage response and repair by promoting the expression of repair-related genes and facilitating repair processes, thereby maintaining genomic stability during development (Fu et al., 2025). Second, by regulating the stability and translation of stress-responsive transcripts through RNA modification, NAT10 helps cells maintain function under adverse conditions. Collectively, these roles enable NAT10 to support balanced proliferation and differentiation, thereby sustaining tissue homeostasis during embryogenesis and organ formation (Fu et al., 2025).

### 3.6 Cell cycle progression and proliferative capacity

NAT10 plays a key role in cell proliferation by enhancing the stability and translation of cell cycle-related mRNAs, thereby promoting G1-to-S phase transition (Jiang et al., 2023). During development, rapid cell proliferation requires increased protein synthesis, and NAT10 supports this demand by boosting translational capacity. NAT10 also contributes to DNA damage checkpoint control, enabling cell cycle progression while safeguarding genomic integrity (Jiang et al., 2023). Importantly, NAT10 catalyzes ac^4^C modification on transcripts of the CCR4-NOT complex, regulating maternal mRNA poly(A) tail decay and precisely controlling the timing of meiosis I progression, oocyte growth, and maturation in mouse oocytes. This study reveals that NAT10 shapes the maternal transcriptome to maintain meiosis and oocyte maturation during oogenesis (Jiang et al., 2023).

### 3.7 T cell expansion

T cell expansion refers to the rapid proliferation of T cells in response to antigen stimulation or cytokine signaling, which is necessary to generate sufficient effector cells for an effective immune response (Sun et al., 2025a). This process is accompanied by differentiation into effector or memory T cells and is tightly regulated by signaling pathways, metabolic state, and transcriptional/epigenetic programs (Sun et al., 2025a). Recent work has shown that T cell activation upregulates NAT10, which catalyzes ac^4^C modification on Myc mRNA, significantly enhancing its translational efficiency. This results in rapid MYC protein synthesis and supports explosive T cell proliferation. T cell-specific deletion of Nat10 leads to MYC deficiency, cell cycle arrest, and impaired expansion, thereby weakening antiviral immune responses. These findings suggest that ac^4^C-mediated translational control of key transcription factors is a central mechanism driving T cell expansion (Sun et al., 2025a). NAT10 plays a critical role in immune cell expansion and function through ac4C-mediated regulation of key transcripts. In hematopoietic stem and progenitor cells (HSPCs), NAT10 deposits ac4C on mRNAs encoding essential hematopoietic transcription factors, such as Nfix, enhancing their translation and thereby supporting HSC self-renewal and megakaryocyte-erythroid progenitor differentiation (Huang et al., 2026). Loss of NAT10 disrupts this ac4C-dependent translational control, leading to impaired HSC maintenance and hematopoietic failure. Beyond the stem cell compartment, NAT10 also regulates T cell proliferation and activation (Li et al., 2025b). T cell-specific NAT10 deficiency reduces mature T cell numbers in peripheral lymphoid organs, as NAT10-mediated acetylation of RACK1 at K185 prevents its K48-linked ubiquitination and degradation, stabilizing RACK1 to promote ribosome assembly and cellular metabolism, ultimately supporting T cell proliferation (Li et al., 2022a). These findings indicate that NAT10 orchestrates both hematopoietic and lymphoid cell expansion via ac4C-dependent mechanisms, highlighting its potential impact on immune homeostasis and the effects of NAT10-targeted therapies.

## 4 NAT10 functions and mechanisms in cancer progression

**FIGURE 2:**
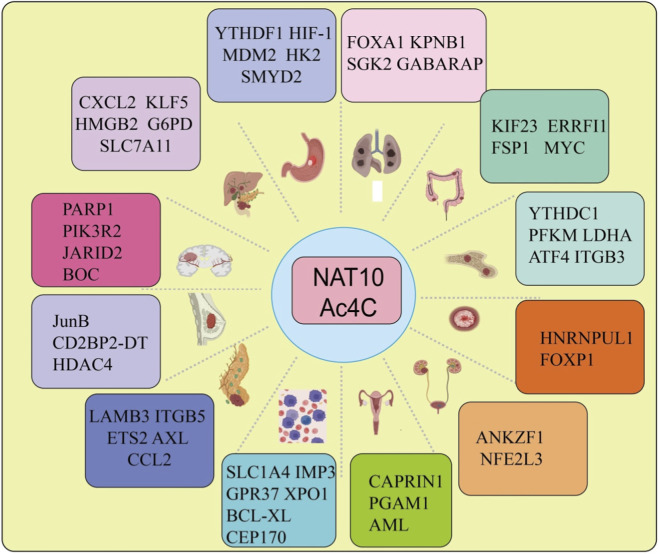
Pan-cancer landscape of NAT10-mediated ac4C modification and its downstream targets. Schematic illustration summarizing the dysregulated expression of NAT10 and its ac4C-modified downstream targets across multiple cancer types. NAT10-mediated ac4C modification regulates diverse oncogenic pathways, including metabolic reprogramming, cell proliferation, invasion and metastasis, apoptosis resistance, and immune regulation, through modifying key mRNAs involved in tumor initiation and progression. Representative target genes identified in different tumor contexts are highlighted, underscoring the tissue-specific and context-dependent roles of NAT10-driven ac4C modification in cancer.

To date, accumulating evidence has revealed that NAT10-mediated ac4C modification is widely dysregulated across multiple cancer types and participates in diverse malignant phenotypes through distinct downstream targets. In Figure 2, we provides an overview of the pan-cancer regulatory landscape of NAT10–ac4C signaling and summarizes representative ac4C-modified genes involved in tumor progression in different tissues.

### 4.1 Digestive system cancers

#### 4.1.1 Hepatocellular carcinoma (HCC)

HCC is the most common primary liver cancer and often develops in the context of chronic liver disease (Ioannou, 2021). In metastatic gastric cancer cells, NAT10 is aberrantly upregulated and catalyzes ac^4^C modification on CXCL2 and KLF5 mRNAs, enhancing their stability. This promotes adhesion between tumor cells and hepatocytes and facilitates metastatic colonization (Chen et al., 2025b). Moreover, CXCL2 induces M2-like macrophages to secrete tumor-promoting factors, which activate STAT3 and further upregulate NAT10, forming a positive feedback loop that links tumor, immune microenvironment, and metastasis (Chen et al., 2025b). In HCC, both NAT10 and ac^4^C levels are elevated; NAT10 catalyzes ac^4^C in the coding sequence of HMGB2 mRNA, enhancing eEF2 binding and promoting HMGB2 translation, thereby driving proliferation and metastasis through a NAT10–ac^4^C/eEF2–HMGB2 axis (Liu et al., 2024a). Upon DNA double-strand breaks (DSBs), NAT10 rapidly accumulates at damage sites and, in a PARP1-dependent manner, acetylates RNA within DNA:RNA hybrids, stabilizing these structures and promoting homologous recombination (HR) repair (Liu et al., 2022). Loss of NAT10 suppresses HCC progression and sensitizes cells to PARP inhibitors, reversing PARP inhibitor resistance in ovarian and breast cancers. In pediatric hepatoblastoma, NAT10 is significantly upregulated; its inhibition suppresses tumor growth and metastasis. Mechanistically, YAP1 binds the NAT10 promoter to increase its transcription, and NAT10 catalyzes ac^4^C modification in the 3′UTR of G6PD mRNA, enhancing its stability and activating the pentose phosphate pathway, thereby driving hepatoblastoma progression (Wang et al., 2025c). In HCC and chemoresistant cells, NAT10 is elevated and associated with poor prognosis. Chemotherapy induces NAT10 relocalization from the nucleolus to the nucleoplasm, where it binds ACLY and acetylates it at K468, preventing SQSTM1-mediated degradation (Wang et al., 2024a). Accumulated nuclear ACLY increases nuclear acetyl-CoA, thereby enhancing H3K27 acetylation and activating transcription of CYP2C9 and PIK3R1, ultimately promoting chemoresistance (Wang et al., 2024a). Additionally, NAT10 upregulation in HCC correlates with poor prognosis and transarterial chemoembolization (TACE) resistance. NAT10 stabilizes SLC7A11 mRNA via ac^4^C, maintaining SLC7A11/GPX4 expression and inhibiting ferroptosis, thus promoting proliferation and metastasis. In contrast, NAT10 silencing induces ferroptosis and suppresses HCC progression (Wang et al., 2025d). Collectively, these findings indicate that NAT10 promotes HCC growth, metastasis, DNA repair, and therapeutic resistance through multiple ac^4^C-dependent mechanisms. In HCC, NAT10 drives tumor growth, metastasis, DNA repair, and treatment resistance mainly by stabilizing oncogenic mRNAs and regulating stress responses via ac^4^C modification.

#### 4.1.2 Gastric cancer

Gastric cancer arises from the gastric mucosal epithelium and is frequently associated with chronic inflammation and *Helicobacter pylori* infection (Karimi et al., 2014). NAT10 expression is elevated in gastric cancer and forms liquid–liquid phase separation (LLPS) condensates; its C-terminal intrinsically disordered region is essential for this tumor-promoting function. NAT10 interacts with the splicing factor SRSF2 and stabilizes it through acetylation, thereby promoting SRSF2-mediated exon 4 skipping in YTHDF1 pre-mRNA and generating a short transcript that enhances proliferation and migration (Liu et al., 2024b). Under hypoxia, HIF-1α upregulates NAT10, which then acetylates SEPT9 mRNA, activating the HIF-1 pathway and reprogramming glucose metabolism (Yang et al., 2023). This establishes a NAT10–SEPT9–HIF-1α positive feedback loop that enhances glycolysis, improves hypoxia tolerance, and promotes resistance to anti-angiogenic therapy. Moreover, both ac^4^C modification and NAT10 are upregulated in gastric cancer and correlate with disease progression and poor prognosis. NAT10 stabilizes MDM2 mRNA via ac^4^C, leading to MDM2 upregulation and p53 downregulation, thereby promoting G2/M progression, proliferation, and tumorigenesis (Deng et al., 2023). *Helicobacter pylori* infection can induce NAT10 expression, triggering an Hp–NAT10–MDM2–p53 signaling axis. In addition, NAT10 and ac^4^C levels correlate with poor prognosis and ^18F^-FDG uptake; NAT10 catalyzes ac^4^C at the junction of the coding sequence and 3′UTR of HK2 mRNA, enhancing its stability and activating glycolysis (Wang et al., 2025e). Conversely, glucose deprivation activates autophagy–lysosomal degradation, increasing NAT10 binding to the SQSTM1/LC3 complex and reducing ac^4^C levels. Clinically, plasma neutrophil extracellular traps (NETs) are elevated in metastatic gastric cancer patients (Wang et al., 2025e); NETs promote proliferation, migration, invasion, and liver metastasis by activating NAT10, which acetylates SMYD2 mRNA and increases its stability. In gastric cancer, NAT10 enhances tumor progression, metabolic reprogramming, and therapy resistance mainly by stabilizing oncogenic transcripts and engaging feedback loops through ac^4^C modification.

#### 4.1.3 Esophageal cancer

Esophageal cancer is a highly aggressive malignancy originating from the esophageal epithelium and is often associated with poor prognosis due to early metastasis (Domper Arnal et al., 2015). In metastatic esophageal cancer, NAT10 undergoes lysine 2-hydroxyisobutyrylation (Khib), particularly at K823, which enhances its interaction with the deubiquitinase USP39 and increases protein stability (Liao et al., 2023). Stabilized NAT10 then enhances NOTCH3 mRNA stability in an ac^4^C-dependent manner, driving metastasis and revealing a novel mechanism of cooperation between protein Khib and RNA acetylation. In esophageal cancer, NAT10 is significantly upregulated and promotes tumor progression by maintaining ac^4^C-modified tRNA levels, thereby increasing the translational efficiency of mRNAs enriched in corresponding codons; EGFR has been identified as a key downstream effector of this tRNA ac^4^C–translation axis (Wei et al., 2023). Additionally, NAT10 enhances translation of ATP6V0E1 mRNA via ac^4^C, increasing v-ATPase activity and lysosomal acidification, which accelerates E-cadherin degradation and promotes metastasis. The interaction between NAT10 and USP39 is also essential for stabilizing ATP6V0E1 expression and lysosomal function. In esophageal cancer (Zhan et al., 2025), NAT10 promotes metastasis and tumor progression through ac^4^C-dependent regulation of both mRNA stability and translational control.

#### 4.1.4 Colorectal cancer

Colorectal cancer (CRC) originates from the epithelial lining of the colon or rectum and is driven by complex genetic and epigenetic alterations (Haraldsdottir et al., 2014). NAT10 is upregulated in CRC and enhances ac^4^C modification, stabilizing KIF23 mRNA via the 3′UTR and leading to KIF23 protein elevation. This activates the Wnt/β-catenin pathway, promoting proliferation, migration, invasion, and liver/lung metastasis; additionally, GSK-3β forms a feedback loop to regulate NAT10 expression (Jin et al., 2022). In another context, NAT10 is highly expressed and promotes tumor progression by binding ERRFI1 mRNA coding region in an ac^4^C-dependent manner, stabilizing ERRFI1 and thereby inhibiting EGFR signaling while maintaining PI3K-AKT pathway inactivity. When NAT10 is inhibited or degraded via UBR5 under 5-fluorouracil treatment (Zheng et al., 2025), ERRFI1 decreases and EGFR signaling is reactivated, constituting a compensatory feedback mechanism that limits the tumor-suppressive effect of NAT10 inhibition. In colon cancer, NAT10 is significantly upregulated and associated with shorter survival; NAT10 stabilizes FSP1 mRNA via ac^4^C, suppressing ferroptosis and promoting proliferation, migration, invasion, tumorigenesis, and metastasis (Zheng et al., 2022). In addition, NAT10 is upregulated under vitamin D receptor (VDR) regulation and enhances translation of PPAN and MYC mRNAs via its acetyltransferase activity (Wang et al., 2026). NAT10-mediated ac^4^C modification at specific sites in PPAN promotes MYC translation, and MYC reciprocally activates PPAN transcription. With MYBBP1A acting as an ac^4^C reader, this forms a NAT10–MYC–PPAN positive feedback axis that drives malignancy and DNA damage repair (Wang et al., 2026). In colorectal cancer, NAT10 drives tumor progression by stabilizing oncogenic mRNAs, modulating key signaling pathways, and suppressing ferroptosis through ac^4^C-dependent mechanisms.

#### 4.1.5 Pancreatic cancer

Pancreatic ductal adenocarcinoma (PDAC) is a lethal malignancy with rapid progression and early metastasis (Klein, 2021). NAT10 is upregulated in PDAC and correlates with poor progression-free survival. It stabilizes LAMB3 mRNA via ac^4^C modification, activating the FAK/ERK pathway, promoting tumor growth, and upregulating PD-L1 expression. This increases the proportion of exhausted CD8^+^ T cells and impairs cytotoxic T cell function in the tumor microenvironment (Chen et al., 2025c). In PDAC with perineural invasion (PNI), ac^4^C modification is significantly elevated, and NAT10 overexpression enhances PNI. Mechanistically, NAT10 catalyzes ac^4^C modification in the coding sequence of ITGB5 mRNA, increasing its stability and activating the ITGB5–pFAK–pSrc adhesion signaling pathway (Huang et al., 2025). In pancreatic cancer, NAT10 overexpression is associated with poor prognosis; it stabilizes ETS2 mRNA via ac^4^C, forming a positive feedback loop that upregulates PD-L1 and suppresses CD8^+^ T cell infiltration, thereby promoting immune evasion (Wang et al., 2025f). NAT10 also stabilizes KRT8 mRNA through ac^4^C, driving proliferation and metastasis (Li et al., 2024b). Moreover, NAT10 mRNA and protein are elevated in PDAC and correlate with poor outcomes; NAT10 enhances the stability of AXL receptor tyrosine kinase mRNA via ac^4^C, promoting proliferation and metastasis (Zong et al., 2023). In cholangiocarcinoma (intrahepatic cholangiocarcinoma, ICC), NAT10 is highly expressed and associated with adverse clinical features. NAT10 binds CCL2 mRNA and increases its expression, leading to CCL2 accumulation in the extracellular matrix, which promotes ICC cell proliferation and induces macrophage polarization toward an M2 phenotype. In pancreatic and biliary cancers (Cai et al., 2024), NAT10 promotes tumor growth, metastasis, immune evasion, and microenvironment remodeling through widespread ac^4^C-dependent stabilization of oncogenic transcripts.

### 4.2 Respiratory system cancers

#### 4.2.1 Lung cancer

Non-small cell lung cancer (NSCLC) is the most common subtype of lung cancer and is often associated with poor prognosis due to early metastasis and therapy resistance (Bade and Dela Cruz, 2020). In cisplatin-resistant NSCLC, NAT10 is highly expressed and stabilizes TRIM44 mRNA in an ac^4^C-dependent manner, thereby activating the PI3K/AKT pathway, upregulating MDR1, and promoting proliferation, invasion, and stemness, which collectively enhance drug resistance (Sun et al., 2025b). Inhibition of NAT10 attenuates TRIM44/PI3K/AKT signaling and restores cisplatin sensitivity. NAT10 is significantly upregulated in NSCLC and correlates with advanced tumor stage and poor survival. Its expression is transcriptionally activated by c-Myc, and in turn promotes Cyclin D1 expression to drive G1/S progression, thereby enhancing proliferation and migration (Wang et al., 2022a). FOXA1 is also upregulated in NSCLC and forms a positive feedback loop with NAT10: NAT10-mediated ac^4^C modification stabilizes FOXA1 mRNA, while FOXA1 acts as a transcription factor to activate NAT10, jointly promoting proliferation, migration, invasion, stemness, and apoptosis resistance (Zhu et al., 2025a). In radioresistant NSCLC cells, NAT10 and ac^4^C levels are elevated; NAT10 acetylates KPNB1 mRNA to increase its expression, which facilitates nuclear translocation of PD-L1 and enhances immune evasion and proliferation, thereby driving radiotherapy resistance (Zhu et al., 2025b). NAT10 inhibition reduces KPNB1/PD-L1 nuclear translocation, restores immune function, and suppresses growth of resistant cells. Another study revealed that NAT10 is markedly upregulated in NSCLC and predicts poor prognosis. NAT10 stabilizes SGK2 mRNA by ac^4^C modification of its 3′UTR, promoting SGK2 interaction with EZH2 and phosphorylation of EZH2 at Thr367 (Xiao et al., 2025). This inhibits EZH2 ubiquitination and increases its stability, while EZH2-mediated H3K27 trimethylation (H3K27me3) suppresses GABARAP transcription, blocking autophagosome–lysosome fusion. Collectively, the NAT10–SGK2–EZH2 axis drives lung cancer proliferation and migration while inhibiting autophagy (Xiao et al., 2025). In lung cancer, NAT10 promotes therapy resistance, proliferation, metastasis, and immune escape primarily through ac^4^C-dependent stabilization of oncogenic transcripts and activation of multiple signaling pathways.

### 4.3 Urinary system cancers

#### 4.3.1 Renal cell carcinoma (RCC)

Clear cell renal cell carcinoma (ccRCC) is the most common subtype of RCC and is characterized by metabolic reprogramming and high metastatic potential (Bahadoram et al., 2022). In ccRCC, ac^4^C modification and NAT10 are upregulated, promoting tumor progression and lymphangiogenesis. Mechanistically, NAT10 stabilizes ANKZF1 expression via ac^4^C modification, and ANKZF1 binds YWHAE to competitively prevent cytoplasmic retention of YAP1, thereby enhancing nuclear translocation of YAP1 and activating transcription of lymphangiogenic genes (Miao et al., 2024). NAT10 is transcriptionally activated by HIF-1α in ccRCC, promoting proliferation, migration, and metastasis. NAT10 stabilizes NFE2L3 mRNA through ac^4^C modification, leading to NFE2L3 upregulation and transcriptional activation of LASP1, which subsequently activates the AKT/GSK3β/β-catenin pathway to drive tumor progression (Sun et al., 2025c). Another study indicated that NAT10 enhances malignant phenotypes in ccRCC while suppressing ferroptosis-related markers, potentially through regulation of the NFE2L1–GPX4 signaling axis, thereby supporting tumor survival and progression (Tan et al., 2025). In ccRCC, NAT10 promotes tumor growth, metastasis, lymphangiogenesis, and ferroptosis resistance via ac^4^C-dependent stabilization of key regulatory transcripts.

#### 4.3.2 Bladder cancer

In bladder cancer, NAT10 catalyzes ac^4^C modification on oncogenic mRNAs such as BCL9L, SOX4, and AKT1, maintaining their stability and translation efficiency. This promotes proliferation, migration, invasion, survival, and stem-like properties of tumor cells, whereas NAT10 depletion reduces ac^4^C peaks on these transcripts and inhibits tumor growth (Wang et al., 2022b). NAT10 also stabilizes AHNAK mRNA through ac^4^C modification, thereby enhancing DNA damage repair and inducing cisplatin resistance. Cisplatin stimulates NF-κB/p65 to upregulate NAT10, forming a positive feedback loop that drives resistance; the NAT10 inhibitor Remodelin can reverse this phenotype (Xie et al., 2023b). Additionally, CLIC3 interacts with NAT10 and inhibits its ac^4^C “writing” function, leading to reduced ac^4^C modification and stability of p21 mRNA, decreased p21 expression, and release of cell cycle inhibition, thereby promoting proliferation. This mechanism also explains the association between high CLIC3 expression and poor prognosis in bladder cancer (Shuai et al., 2024). In bladder cancer, NAT10 supports malignant growth and chemoresistance by stabilizing oncogenic mRNAs through ac^4^C modification, while its inhibition reverses these effects.

#### 4.3.3 Prostate cancer

NAT10 is significantly upregulated in prostate cancer and correlates with adverse clinicopathological indicators such as grade, stage, and Gleason score. NAT10 enhances stability of HMGA1 and KRT8 mRNAs via ac^4^C modification, thereby promoting cell cycle progression and epithelial–mesenchymal transition (EMT), which drives proliferation and migration (Li et al., 2024b). NAT10 also promotes tumor growth, migration, and invasion through an ac^4^C-dependent increase of CCL25 expression, activating the CCL25/CCR9 axis. This suppresses CD8^+^ T cell recruitment and cytotoxicity, creating an immunosuppressive microenvironment that facilitates tumor progression (Liu et al., 2024c). An ac^4^C-based scoring system constructed from NAT10 downstream targets can predict immune infiltration and response to immunotherapy. The NAT10 inhibitor Remodelin markedly suppresses proliferation, migration, and invasion of prostate cancer cells and reduces tumor growth *in vivo*. Mechanistically, NAT10 participates in DNA replication and is associated with pre-replication complexes and replication origins; Remodelin reduces CDC6 and androgen receptor (AR) expression, blocking DNA replication and inhibiting tumor growth, including in castration-resistant prostate cancer (Ma et al., 2022). In prostate cancer, NAT10 drives tumor growth, metastasis, and immune evasion by stabilizing oncogenic transcripts and supporting DNA replication, while its inhibition suppresses malignancy.

### 4.4 Female reproductive system cancers

#### 4.4.1 Breast cancer

NAT10 enhances JunB mRNA stability via increased ac^4^C modification, leading to upregulation of LDHA and promotion of glycolysis, thereby forming a high-glycolysis and immunosuppressive tumor microenvironment that drives triple-negative breast cancer progression (Li et al., 2024c). NAT10 depletion or inhibition by Remodelin enhances T cell activation and, when combined with anti-CTLA-4 antibody, further strengthens antitumor immune responses to suppress tumor growth. CD2BP2-DT is highly expressed in breast cancer and promotes proliferation; its stability is increased by NAT10-mediated ac^4^C modification. CD2BP2-DT promotes YBX1 phase separation to stabilize CDK1 mRNA, thereby activating a CDK1-driven proliferation program (Wang et al., 2025b). NAT10 increases expression and stability of ABC transporters such as MDR1 and BCRP via ac^4^C modification, promoting proliferation, invasion, and chemoresistance; NAT10 inhibition reduces MDR1/BCRP levels and reverses capecitabine resistance (Zhao et al., 2024). NAT10 maintains ac^4^C modification on chromatin-associated tRNAs through its RNA acetylation activity, ensuring p300/CBP function and enhancer activity, thereby sustaining expression of a gene program that promotes metastasis (Amin et al., 2025). NAT10 deficiency induces chromatin remodeling and changes in immune-regulatory gene expression, reducing breast cancer metastasis and inhibiting recruitment of pro-metastatic myeloid cells. NAT10 stabilizes HDAC4 mRNA via ac^4^C modification, and HDAC4 maintains NAT10 protein levels through deacetylation, forming a self-reinforcing loop (Ma et al., 2026). This loop activates NF-κB signaling and increases PD-L1 expression, promoting immune evasion and tumor progression in breast cancer.

#### 4.4.2 Cervical cancer

NAT10 is upregulated in cervical cancer and correlates with poor prognosis. It enhances HNRNPUL1 mRNA stability through ac^4^C modification, increasing HNRNPUL1 expression and promoting proliferation, migration, and invasion. HNRNPUL1 depletion inhibits these malignant phenotypes, indicating that the NAT10–ac^4^C–HNRNPUL1 axis is a key driver of cervical cancer progression (Long et al., 2023). After being transcriptionally activated by HOXC8, NAT10 enhances FOXP1 mRNA translation efficiency via ac^4^C modification, leading to upregulation of GLUT4 and KHK and promoting glycolysis with sustained lactate production. Lactate accumulation in the tumor microenvironment strengthens Treg-mediated immunosuppression, forming an immune escape mechanism, and NAT10 inhibition enhances the efficacy of anti–PD-L1 therapy (Chen et al., 2023).

#### 4.4.3 Ovarian cancer

NAT10 is highly expressed in ovarian cancer cells and promotes proliferation, migration, invasion, stemness maintenance, and EMT in an ac^4^C-dependent manner by modifying CAPRIN1 mRNA. NAT10 increases CAPRIN1 ac^4^C levels to stabilize its transcript, leading to CAPRIN1 upregulation and driving malignant phenotypes and tumor growth (Song and Cheng, 2025). NAT10 rapidly accumulates at DNA double-strand break (DSB) sites and, in a PARP1-dependent manner, acetylates RNA in DNA:RNA hybrids to enhance hybrid stability and promote homologous recombination repair; NAT10 depletion inhibits tumor progression. Structural studies show that NAT10 binds Remodelin to form a C2-symmetric conformation, and NAT10 inhibition increases sensitivity to PARP inhibitors (PARPi) and reverses PARPi resistance in ovarian and breast cancer, suggesting that combined NAT10/PARP1 inhibition may represent a novel synthetic lethality strategy (Xu et al., 2025). NAT10 is highly expressed in ovarian cancer and enhances mRNA ac^4^C modification to stabilize and upregulate the glycolytic enzyme PGAM1, thereby promoting metabolic reprogramming and stemness maintenance and driving tumor growth; PGAM1 restoration reverses the tumor-suppressive effects of NAT10 loss, indicating that the NAT10–PGAM1 axis is a core metabolic driver in ovarian cancer (Zhang and Shen, 2025).

### 4.5 Nervous system tumors

DPT reduces intracellular NAD^+^ levels, activates PARP1 to induce parthanatos in glioma cells, and amplifies this death signal by upregulating NOX2 to trigger ROS-dependent DNA double-strand breaks and by enhancing PARP1 acetylation through NAT10 (Liang et al., 2023). Meanwhile, JNK-mediated phosphorylation activates SIRT1, forming a JNK–SIRT1 positive feedback loop that further promotes NOX2 and NAT10 expression, thereby driving parthanatos (Liang et al., 2023). NAT10 is highly expressed in glioblastoma (GBM) and correlates with malignant pathological features and poor prognosis; it stabilizes PIK3R2 mRNA via ac^4^C modification, promoting proliferation, migration, and invasion, thus driving GBM progression (Meng et al., 2025). NAT10 is upregulated in GBM and associated with poor prognosis; it enhances JARID2 mRNA stability and protein expression through ac^4^C modification, activating PRC2-related epigenetic regulation to maintain tumor stemness and promote malignant progression (Inoki et al., 2025). NAT10 is upregulated in GBM and promotes proliferation and migration by enhancing stability and translation efficiency of BOC mRNA via ac^4^C modification. Under hypoxic conditions (Lin et al., 2026), HIF1α directly activates NAT10 transcription, strengthening ac^4^C modification function and forming a “hypoxia–NAT10–BOC” axis that drives GBM malignancy.

### 4.6 Hematologic and lymphoid tumors

#### 4.6.1 Acute myeloid leukemia (AML)

NAT10 catalyzes ac^4^C modification to enhance translation of SLC1A4 and upregulate HOXA9/MENIN, thereby activating the serine synthesis pathway and reprogramming serine metabolism in AML cells to maintain leukemia stem/progenitor cell self-renewal and leukemogenic capacity; NAT10 inhibition disrupts this metabolic dependency and significantly suppresses AML growth (Zhang et al., 2024d). Venetoclax reduces NAT10 expression and NAT10-mediated ac^4^C modification in AML, decreasing IMP3 mRNA stability and expression, thereby promoting apoptosis and inhibiting cell viability (Lu et al., 2025); restoration of NAT10 or IMP3 reverses this effect, suggesting that venetoclax’s anti-AML activity partly depends on the NAT10–IMP3 axis.

#### 4.6.2 Multiple myeloma

NAT10 acetylates GPR37 mRNA via ac^4^C modification to increase its expression, promoting proliferation, cell cycle progression, glycolysis, and immune evasion while inhibiting apoptosis in multiple myeloma cells; NAT10 inhibition reverses these malignant phenotypes and suppresses tumor growth by downregulating GPR37 (Liu et al., 2025). NAT10 is upregulated in multiple myeloma and stabilizes XPO1 mRNA via ac^4^C modification, promoting resistance to proteasome inhibitors such as bortezomib (BTZ) (Xu et al., 2024); NAT10 inhibition or combined XPO1 inhibition significantly reverses resistance and enhances therapeutic efficacy. NAT10 is upregulated in multiple myeloma and stabilizes and enhances translation of BCL-XL mRNA through ac^4^C modification, inhibiting apoptosis; it also activates the PI3K–AKT pathway and upregulates CDK4/6 to promote proliferation, and NAT10 inhibition reverses these malignant phenotypes (Zhang et al., 2022). NAT10 is upregulated in multiple myeloma and promotes tumor growth by enhancing translation efficiency of CEP170 mRNA via ac^4^C modification, driving proliferation and chromosomal instability; NAT10 inhibition (e.g., Remodelin) suppresses tumor growth and prolongs survival in mouse models (Wei et al., 2022).

### 4.7 Osteosarcoma

NAT10 maintains stability and translation of the m^6^A reader YTHDC1 via ac^4^C modification, which in turn regulates stability of PFKM and LDHA mRNAs in an m^6^A-dependent manner, promoting glycolysis and proliferation/migration in osteosarcoma (Mei et al., 2024); NAT10 inhibition reduces YTHDC1 levels, increases m^6^A content, and suppresses tumor growth and invasion, revealing a key NAT10/ac^4^C–YTHDC1/m^6^A–glycolysis axis (Mei et al., 2024). NAT10 enhances ATF4 mRNA stability via ac^4^C modification, upregulating ASNS transcription and promoting asparagine synthesis to drive osteosarcoma growth and progression; targeting NAT10 (e.g., paliperidone, AG-401) inhibits osteosarcoma progression and shows synergistic antitumor effects in patient-derived xenograft (PDX) models (Zou et al., 2024). NAT10 is highly expressed in osteosarcoma and upregulates ITGB3 mRNA via ac^4^C modification, promoting proliferation, migration, and invasion; its transcription is activated by YY1, which cooperates with NAT10 to further enhance ITGB3 expression, forming a YY1–NAT10–ITGB3 axis that drives osteosarcoma progression (Yang and Wang, 2025).

### 4.8 Head and neck tumors

NAT10 is highly expressed in nasopharyngeal carcinoma (NPC) and enhances stability and translation efficiency of CEBPG, DDX5, and HLTF mRNAs via ac^4^C modification. DDX5-dependent upregulation of HMGB1 suppresses CD4^+^/CD8^+^ T cell infiltration (Xie et al., 2025), creating an immunosuppressive microenvironment; HLTF reciprocally transcriptionally activates NAT10, forming an HLTF–NAT10 positive feedback loop that promotes tumor progression and influences PD-1 therapy sensitivity (Xie et al., 2025). NAT10 is upregulated in NPC and promotes SLC7A11 expression via ac^4^C modification, thereby inhibiting sorafenib-induced ferroptosis and causing drug resistance; NAT10 inhibition decreases SLC7A11 and enhances sorafenib sensitivity, revealing the critical role of the NAT10–ac^4^C–SLC7A11 axis in sorafenib resistance (Xue et al., 2024). NAT10 enhances stability of lncRNA SIMALR via ac^4^C modification, leading to its overexpression in NPC; SIMALR promotes eEF1A2 GTPase activity and phosphorylation, strengthening translation of ITGB4/ITGA6 and driving NPC proliferation and metastasis, highlighting a NAT10–SIMALR–eEF1A2–integrin axis in protein translation regulation and tumor progression (Gong et al., 2024). In laryngeal squamous cell carcinoma (LSCC), NAT10 is upregulated and enhances FOXM1 mRNA stability and expression through ac^4^C modification of the 3′UTR, promoting proliferation, migration, and invasion and accelerating tumor growth *in vivo* (Li et al., 2023). Mechanistically, NAT10-dependent ac^4^C modification activates the FOXM1 signaling pathway, suggesting that blocking the NAT10–ac^4^C–FOXM1 axis may be a potential therapeutic strategy for LSCC.

### 4.9 NAT10-mediated ac^4^C regulation of EMT and cancer metastasis

Epithelial–mesenchymal transition (EMT) is a fundamental process in cancer progression, during which epithelial cells acquire mesenchymal traits, enhancing migratory and invasive capacities and contributing to metastasis and therapy resistance (Manfioletti and Fedele, 2023). Accumulating evidence indicates that NAT10 promotes EMT and metastasis through ac^4^C-dependent stabilization of key transcripts. In prostate cancer, NAT10 enhances the stability of KRT8 mRNA, thereby facilitating EMT and promoting tumor cell migration, while stabilization of HMGA1 mRNA advances cell cycle progression and proliferation (Li et al., 2024b). In gastric and non-small cell lung cancers, NAT10 upregulation correlates with poor prognosis, and its knockdown reduces EMT marker expression, including decreased N-cadherin and vimentin and increased E-cadherin (Guo et al., 2024; Song et al., 2024). In breast cancer and hepatocellular carcinoma, NAT10 inhibition by Remodelin or siRNA reverses EMT, diminishes chemoresistance, and suppresses hypoxia-induced EMT, demonstrating its functional importance in metastasis and therapy response (Wu et al., 2018). Collectively, these studies establish NAT10 as a pro-metastatic regulator that drives EMT by stabilizing transcripts of cytoskeletal and transcriptional regulators, highlighting NAT10 as a potential therapeutic target to impede cancer invasion and dissemination.

### 4.10 NAT10 in cancer therapy resistance

Therapeutic resistance is a major barrier in cancer treatment, arising from diverse mechanisms such as enhanced DNA repair, dysregulated cell cycle checkpoints, evasion of apoptosis, and altered drug metabolism or transport (Wei et al., 2023). Accumulating evidence indicates that NAT10 contributes to resistance against multiple chemotherapeutic and targeted agents through ac^4^C-mediated stabilization of key mRNAs and post-translational regulation. In bladder cancer, NAT10 enhances cisplatin resistance by stabilizing AHNAK mRNA, promoting DNA damage repair, while NFκB signaling upregulates NAT10 in response to cisplatin (Xie et al., 2023b). In hepatocellular carcinoma and breast or ovarian cancers, NAT10 facilitates resistance to PARP inhibitors by stabilizing RNA at DNA:RNA hybrids and promoting homologous recombination; pharmacological inhibition with Remodelin restores PARPi sensitivity (Xu et al., 2025). NAT10 also confers resistance to doxorubicin in breast and hepatocellular carcinoma by modulating EMT through Twist-dependent pathways. In nasopharyngeal carcinoma, NAT10-mediated ac^4^C acetylation of SLC7A11 mRNA suppresses sorafenib-induced ferroptosis, and NAT10 inhibition enhances drug sensitivity (Wang et al., 2025d). Additionally, NAT10 promotes cisplatin resistance in gastric cancer via activation of the Wnt/β-catenin pathway, and drives EGFR-TKI resistance in NSCLC by stabilizing fatty acid metabolism regulators (FATP4, CPT1A) (Fang et al., 2025). Collectively, these studies establish NAT10 as a central epitranscriptomic regulator of therapeutic resistance, acting through mRNA stabilization, DNA repair enhancement, EMT regulation, and metabolic remodeling, highlighting its potential as a target to overcome chemoresistance and improve cancer treatment outcomes.

## 5 Diagnostic and therapeutic potential of NAT10 in cancer

### 5.1 NAT10 as a diagnostic biomarker in cancer

NAT10, the primary “writer” of RNA N4-acetylcytidine (ac^4^C), is broadly overexpressed across multiple cancer types and closely associated with tumor proliferation, invasion, therapeutic resistance, and poor prognosis, indicating significant clinical translational potential (Yang et al., 2021). A growing body of clinical evidence demonstrates that NAT10 is upregulated in both solid and hematological malignancies, and its expression level correlates with tumor stage, differentiation grade, lymph node metastasis, and patient survival (Yang et al., 2021). Notably, in some cancers, NAT10 exhibits subcellular relocalization from the nucleolus/nucleus to the cytoplasm or membrane, which is often linked to enhanced invasiveness and worse outcomes. This suggests that NAT10 may serve not only as a quantitative biomarker but also as an indicator of tumor progression based on its cellular distribution (Li et al., 2024d). Moreover, emerging studies suggest that NAT10 or ac^4^C-related downstream molecules may be detectable in bodily fluids such as blood or exosomes, offering potential for non-invasive diagnosis and dynamic monitoring. Collectively, NAT10 represents a biomarker reflecting the “epitranscriptomic activity” of tumors and may aid in diagnosis, risk stratification, and prediction of treatment response. In papillary renal cell carcinoma (pRCC) and several other cancers (Yang et al., 2021; Li et al., 2024d), NAT10 is broadly upregulated and associated with advanced stage, poor prognosis, and immune microenvironment dysregulation; prognostic models based on NAT10-related genes demonstrate high predictive accuracy and indicate that high-risk patients may respond poorly to immunotherapy. In HCC, NAT10 correlates positively with various immune infiltrating cells, including T cells, macrophages, and dendritic cells, as well as immune-related gene signatures, suggesting that NAT10 may influence tumor progression through modulation of the immune microenvironment (Li et al., 2017). Accumulating evidence suggests that NAT10 may serve as a potential diagnostic and prognostic biomarker across multiple cancer types. NAT10 expression is frequently upregulated in tumor tissues, including AML, HCC, and COAD, and its elevated levels are associated with adverse clinicopathological features and poor patient outcomes. For instance, in AML, high NAT10 mRNA expression correlates with NPM1 mutation status, lower remission rates, and reduced progression-free and overall survival (Liang et al., 2020). In HNSC, NAT10 protein is predominantly localized in nuclei/nucleoli, and high expression correlates with advanced TNM stage, poor histological differentiation, and serves as an independent prognostic factor (Tao et al., 2021). Beyond tissue-based analyses, NAT10-mediated ac^4^C modifications in circulating RNA or tumor-derived transcripts may provide a noninvasive avenue for liquid biopsy-based detection, although further validation is needed. Collectively, these findings support the utility of NAT10 expression and ac^4^C signatures as predictive biomarkers, offering potential guidance for prognosis assessment and personalized therapy selection.

### 5.2 Therapeutic potential and limitations of targeting NAT10

Targeting NAT10 holds clear anti-tumor potential. Since NAT10 enhances the stability and translation of numerous oncogenes and proliferation-related mRNAs via ac^4^C modification, its inhibition could simultaneously disrupt multiple oncogenic pathways, conferring a “multi-target” therapeutic advantage (Zou et al., 2024). Additionally, because NAT10 participates in DNA damage repair, metabolic reprogramming, and immune evasion, its inhibition may sensitize tumors to chemotherapy and radiotherapy and reverse drug resistance (Zou et al., 2024). By suppressing tumor proliferation and metabolic adaptability, NAT10 inhibition may also remodel the tumor immune microenvironment, potentially synergizing with immunotherapy. Pharmacological inhibition of NAT10 represents a promising therapeutic avenue in cancer, with Remodelin being the most widely studied inhibitor. Beyond Remodelin, other small molecules, including FDA-approved drugs such as fludarabine and fosaprepitant, have demonstrated potent anti-leukemia activity by targeting NAT10, with IC50 values substantially lower in AML cells than in normal peripheral blood mononuclear cells (Zhang et al., 2024d). These findings highlight the potential for drug repurposing to achieve NAT10 inhibition. Nevertheless, current NAT10-targeting strategies face several challenges, including limited specificity, potential off-target effects, toxicity, and efficient delivery to tumor sites (Zhang et al., 2024d). Efforts are ongoing to develop more selective small molecules, PROTACs, or optimized formulations to mitigate off-target toxicity and improve bioavailability. Addressing these issues will be crucial for translating NAT10-targeted therapies into clinical applications and realizing their full therapeutic potential.

However, several significant challenges remain. NAT10 plays crucial roles in normal development, stem cell self-renewal, and immune cell expansion; systemic inhibition may therefore cause adverse effects such as myelosuppression, impaired immune function, or tissue regeneration defects (Zou et al., 2024). Existing NAT10 inhibitors (e.g., Remodelin) also face limitations in specificity, pharmacokinetics, and tissue distribution. Achieving effective tumor suppression while minimizing effects on normal tissues remains a key obstacle (Zhang et al., 2024d). Moreover, tumor cells may evade NAT10 inhibition through compensatory mechanisms, such as alternative modifying enzymes, downstream pathway activation, or metabolic reprogramming, indicating that combination therapies or more precise intervention strategies are needed.

In summary, despite its substantial anti-tumor potential, clinical translation of NAT10 targeting requires overcoming challenges related to therapeutic window, selectivity, resistance mechanisms, and combination strategies. Future research should focus on developing highly specific NAT10 inhibitors, exploring tumor-targeted delivery systems, and identifying patient stratification biomarkers. Additionally, targeting NAT10 condensates in macrophages can promote macrophage polarization from an immunosuppressive M2 phenotype to a pro-inflammatory M1 phenotype by regulating SRSF2 stability; in colorectal cancer models (Zhu et al., 2026), NAT10 silencing reshapes the tumor immune microenvironment and enhances anti-tumor immunity, thereby suppressing tumor growth. In colorectal cancer, NAT10 stabilizes DKK2 mRNA through ac^4^C modification and promotes its secretion; DKK2 acts on the LRP6 receptor on CD8^+^ T cells to activate AKT–mTOR signaling, inducing cholesterol accumulation and weakening cytotoxic function (Li et al., 2026). NAT10 deficiency enhances CD8^+^ T cell infiltration and anti-tumor immunity, highlighting a NAT10–DKK2–LRP6–AKT–mTOR–cholesterol axis in immune evasion. Furthermore, NAT10 promotes NPM1 acetylation and enhances its transcriptional activity, thereby upregulating PD-L1 expression in various cancers; NAT10 levels correlate positively with PD-L1, indicating a NAT10–NPM1 acetylation–PD-L1 transcription axis in immune suppression (Qin et al., 2024). The tumor-derived lncRNA GAS5 remodels the immune microenvironment in non-small cell lung cancer (NSCLC) by activating type I interferon signaling: GAS5 binds MYBBP1A and promotes its interaction with p53, enhancing IRF1 transcription and inducing CXCL10/CCL5 secretion, which recruits macrophages and T cells and suppresses tumor progression (Wang et al., 2024b). The stability of GAS5 is regulated by NAT10, forming an upstream immune-regulatory mechanism. The NAT10-targeting PROTAC degrader NP1192 markedly reduces NAT10 levels and inhibits ac^4^C modification and translation of HIF1A mRNA, thereby reducing lactate production and ATP supply under hypoxia and suppressing HIF-1α–driven PD-L1 upregulation. *In vitro*, *in vivo*, and in tumor organoid models (Wu et al., 2020), NP1192 combined with anti-PD-L1 enhances CD8^+^ effector T cell activity, reduces exhausted T cells, reverses immune checkpoint blockade resistance, and improves immunotherapy efficacy. RNA ac^4^C modification enhances tumor immune suppression through the NAT10–DDX5–HMGB1 axis, inhibiting CD4^+^ and CD8^+^ T cell function and promoting immune escape in nasopharyngeal carcinoma, leading to anti-PD-1 resistance (Xie et al., 2025). These findings underscore the importance of epitranscriptomic regulation in shaping the tumor immune microenvironment and suggest that NAT10 inhibition may provide a new strategy for combination immunotherapy.

## 6 Discussion and future perspectives

NAT10 is the only known “writer” of RNA N4-acetylcytidine (ac^4^C) and exerts multilayered regulatory functions during tumor initiation and progression. However, several key questions remain unresolved, particularly regarding the mechanisms underlying its aberrant expression across different cancer types, the commonality and specificity of its oncogenic pathways, and how to achieve tumor-selective targeting while preserving its essential physiological roles. First, although NAT10 overexpression has been reported in multiple malignancies, the upstream drivers appear heterogeneous. In some cancers, gene amplification or transcriptional activation may account for NAT10 upregulation, whereas in others, it may be induced by oncogenic signaling pathways (e.g., PI3K/AKT, MYC) or cellular stress responses such as oxidative stress and DNA damage. Additionally, altered subcellular localization of NAT10 (e.g., nucleolus-to-cytoplasm or membrane translocation) may reflect distinct functional requirements in different tumor microenvironments. Therefore, future studies should systematically elucidate the molecular drivers of NAT10 upregulation in specific tumor contexts and clarify its association with genomic background, microenvironmental cues, and therapeutic pressure, which will be essential for precise patient stratification and rational targeting.

Although NAT10 is predominantly described as an oncogenic factor in multiple cancer types, it is conceivable that its function may be context-dependent and could, under certain conditions, exhibit tumor-suppressive effects. The duality of NAT10 activity may be influenced by cell type, genetic background, epigenetic landscape, or the repertoire of RNA and protein substrates available in a given tissue. For example, in cells where NAT10 primarily stabilizes mRNAs of pro-survival or proliferation-related genes, its activity promotes tumor progression, whereas in other contexts, NAT10 could conceivably enhance the stability of mRNAs encoding tumor suppressors or DNA repair factors, thereby restraining malignancy. Recognizing this potential for context-specific roles underscores the importance of mechanistic studies across diverse cellular and genetic settings, and cautions against assuming uniform oncogenicity of NAT10 in all cancers.

Second, NAT10 plays crucial roles in normal development, stem cell self-renewal, and immune cell expansion, and its mechanistic functions largely overlap with those observed in tumors, such as mRNA stabilization, enhanced translation, and increased cell proliferation. This raises concerns that direct systemic inhibition of NAT10 may impair normal tissue homeostasis and lead to adverse effects. To address this challenge, two complementary strategies are warranted. One approach is to develop tumor-specific delivery systems (e.g., nanocarriers, antibody–drug conjugates, or microenvironment-responsive release) to concentrate NAT10 inhibitors in malignant tissues. Another strategy is to identify cancer-specific NAT10 dependencies, such as tumor-restricted ac^4^C target transcripts or unique downstream pathways, enabling more selective intervention with reduced toxicity. Third, NAT10 is involved in proliferation, DNA damage repair, and metabolic reprogramming, suggesting that its inhibition may sensitize tumors to chemo-/radiotherapy and overcome resistance. However, NAT10 also contributes to immune cell expansion, implying that its inhibition could potentially compromise anti-tumor immunity. Therefore, the combination of NAT10 inhibitors with immunotherapy presents a double-edged sword: while reducing tumor immunosuppression and enhancing tumor cell death may potentiate immune responses, suppression of T cell proliferation or function could weaken therapeutic efficacy. Future studies should thus focus on evaluating the impact of NAT10 inhibition on immune cell biology and exploring optimal dosing schedules or localized delivery strategies to maximize tumor suppression while preserving immune competence.

Despite the established role of NAT10 in T cell expansion and antiviral immunity, its functions within the tumor immune microenvironment remain incompletely understood. For instance, whether NAT10 regulates the activity of tumor-infiltrating immune cells (such as T cells, macrophages, and dendritic cells), immune checkpoint expression, cytokine secretion, or immune evasion mechanisms requires systematic investigation. Single-cell transcriptomics, immunohistochemistry, and functional immune assays could help define the ac^4^C landscape in immune cells and reveal how NAT10 shapes anti-tumor immunity, thereby providing a rationale for combinational immunotherapy. Moreover, ac^4^C is primarily regarded as an RNA modification, but its potential influence on chromatin regulation and transcriptional control remains largely unexplored. Since RNA modifications can modulate the stability or function of lncRNAs, miRNAs, or RNA-binding proteins that are involved in chromatin organization, future research should address whether ac^4^C participates in: (1) regulating chromatin-associated lncRNA stability and function; (2) modulating expression or translation of chromatin regulators; and (3) indirectly affecting histone modifications or DNA methylation through RNA-mediated feedback loops. Demonstrating a chromatin-level role for ac^4^C would significantly broaden its relevance in epigenetic regulation and provide new insights into tumorigenesis.

RNA-binding proteins play a central role in modulating RNA fate in cancer, influencing processes such as mRNA stability, splicing, localization, and translation (Schuschel et al., 2020; Khan et al., 2024). Several RBPs have been shown to recognize ac4C-modified transcripts, thereby linking NAT10-mediated acetylation to post-transcriptional regulation. NAT10 catalyzes the only known RNA N4-acetylcytidine (ac^4^C) modification, yet its substrate specificity and enzymatic targeting mechanisms have remained largely unclear. Recent studies have revealed that NAT10 forms a complex with RNA-binding proteins, including poly (rC)-binding proteins 1/2 (PCBP1/2) and TAR DNA-binding protein 43 (TDP43), which serve as adaptors to tether NAT10 to specific mRNAs (Jiang et al., 2024). This NAT10/PCBP/TDP43 complex guides ac^4^C deposition preferentially at cytidine-rich motifs, as knockdown of these adaptors reduces mRNA acetylation and abolishes sequence-specific ac^4^C sites. Functional analyses in HEK293T cells and detection in mouse testes suggest that this complex not only determines substrate selection but also has physiological relevance *in vivo* (Jiang et al., 2024).

Unlike m^6^A modification, which has well-defined writers, readers, and erasers, ac^4^C currently has only a confirmed writer (NAT10), while its “eraser” remains unknown. Identifying an ac^4^C eraser will be critical for understanding the dynamic regulation of this modification and may offer additional therapeutic targets. Future efforts should combine systematic screening, structural biology, and functional validation to identify potential deacetylases or RNA decay-dependent clearance mechanisms and elucidate their roles in physiology and cancer. Similarly, although ac^4^C likely requires specific reader proteins to mediate its effects, ac^4^C readers have not yet been clearly defined. Discovering ac^4^C binding proteins will help explain how ac^4^C influences RNA fate (e.g., stability, translation, splicing, or localization) and provide more precise targets for intervention. Approaches such as RNA affinity purification, mass spectrometry, and CRISPR screening may be employed to systematically identify ac^4^C readers and characterize their structure-function relationships. In summary, NAT10-mediated ac^4^C modification plays a pivotal role in both normal development and tumor progression, with complex and context-dependent mechanisms. Future research should advance along three parallel lines: (1) elucidating the mechanisms of NAT10 upregulation and localization changes, defining ac^4^C target specificity, and exploring crosstalk with other epigenetic layers; (2) developing highly selective and low-toxicity NAT10 inhibitors and evaluating combinational regimens with immunotherapy; and (3) identifying ac^4^C erasers and readers to construct a complete ac^4^C regulatory network. Such efforts will provide a solid foundation for precision intervention and clinical translation.
